# Non-cognitive ability and relative poverty in farmer households: an empirical study from rural China

**DOI:** 10.3389/fpsyg.2025.1558546

**Published:** 2025-04-23

**Authors:** Bochi Sun, Han Yin

**Affiliations:** ^1^School of Economics, Tianjin Normal University, Tianjin, China; ^2^School of Statistics, Tianjin University of Finance and Economics, Tianjin, China

**Keywords:** non-cognitive ability, farmer households’ relative poverty, self-development ability, economic decision-making, pro-social behavior

## Abstract

Exploring the cognitive aspects of pro-social behavior is crucial for improving social well-being and strengthening social identity. Specifically, in today’s post-poverty alleviation era, it is vital to pay attention to how non-cognitive ability can alleviate the relative poverty of farmers and further promote the consolidation and expansion of poverty alleviation achievements from the perspective of pro-social behavior. This study incorporates non-cognitive abilities into a traditional economic model, constructing a theoretical framework to analyze their impact on the economic decision-making of farmer households. Utilizing rural sample data from the 2018 China Family Panel Studies, we employed the instrumental variable and two-stage least squares methods to empirically examine this effect. The main findings of the study are as follows. (1) The empirical test results of Hypothesis 1 indicate that an enhancement in non-cognitive abilities significantly reduces relative poverty, particularly traits such as conscientiousness and extraversion, whereas agreeableness, openness, and emotional stability show no significant impact. Notably, non-cognitive abilities have a more pronounced effect on female-headed and low-educated farmer households in Western China, thereby demonstrating inclusiveness. (2) The empirical test results of Hypothesis 2 indicate that improved non-cognitive abilities enhance the quality of economic decision-making by alleviating constraints, adjusting preferences, and enhancing expectations, thereby reducing relative poverty. To effectively mitigate relative poverty among farmer households, governments must promote and provide training for these non-cognitive abilities, thus bolstering self-development capabilities and improving economic decision-making.

## 1 Introduction

Eradicating poverty remains a long-term challenge amid the economic development of developing countries (Wang et al., 2022). In 2015, the United Nations introduced the Sustainable Development Goals (SDGs), aiming to eliminate all forms of poverty by 2030. China, one of the world’s most dynamic economies, has made significant strides in this area. By the end of 2020, under the existing poverty standards, 98.99 million rural inhabitants, 832 counties, and 128,000 villages had overcome poverty. This achievement marked the end of regional poverty and the completion of the formidable task of eradicating absolute poverty, setting a global precedent as the “China Sample” for poverty alleviation (Meng et al., 2024). However, the goal of poverty eradication extends beyond merely lifting people above the poverty line. In China, a large segment of the rural population, which has recently risen above the absolute poverty threshold, remains vulnerable. Although not considered impoverished by absolute standards, this rural population falls into the category of relative poverty when compared to average household incomes. As poverty standards evolve, these populations risk slipping back into poverty, potentially undermining the progress made and affecting the implementation of the rural revitalization strategy. Thus, the issue of relative poverty among farming households has emerged as a critical concern for both government and academic circles.

Pro-social conduct encompasses actions aligning with societal norms while demonstrating positive impacts on individuals, communities, and broader collectives. It is an important aspect of individual social cognition and emotion. Moreover, it plays an important role in promoting the well-being of others, strengthening social connections and social identity (Li et al., 2018; Ramkissoon, 2020), and promoting individual social adaptation and interpersonal harmony. Prosocial behavior not only affects the individuals’ economic decision-making (Trajano et al., 2023) but also has a close relationship with subjective psychological factors, such as non-cognitive ability (Palczyńska, 2021). Studying pro-social behavior from the perspective of non-cognitive ability facilitates a more comprehensive understanding of the psychological factors underpinning pro-social behavior, such as motivation, emotion, and values, in addition to accurately grasping its essence and changing rules. These advantages can help policymakers formulate more targeted and effective policy measures. Non-cognitive abilities typically include an individual’s personality traits, motivations, ambitions, and social communication skills. Individuals with varying levels of non-cognitive abilities often make different economic decisions even under similar circumstances, impacting their socio-economic activities (Parise and Peijnenburg, 2019). Empirical studies have shown that high non-cognitive ability significantly enhances individuals’ salary income (Almlund et al., 2011; Anghel and Balart, 2017), educational attainment (Buser et al., 2024), health (Smithers et al., 2018), social capital (Li, 2024), and entrepreneurial endeavors (Levine and Rubinstein, 2017). Non-cognitive abilities play a critical role in optimizing socio-economic decision-making for individuals and households, thus contributing significantly to alleviating relative poverty. The relative poverty of farmers reflects a state of inertia: not seeking progress and being content with the present. This state is the main manifestation of the low level of rigor, extroversion, and openness in non-cognitive ability. An improvement of non-cognitive ability can solve the problems of farmers’ ideological dependence and lack of progress in work, and provide lasting development momentum for farmers. Sustainable development momentum is conducive to reducing farmers’ risk and relative poverty.

To date, few studies have quantitatively assessed the impact of non-cognitive abilities on the relative poverty of farming households. Previous research has typically defined relative poverty among these households using established standards—namely, 40 to 50% of the median or mean rural household per-capita net income (Yi et al., 2023) —and analyzed it through the lenses of public policies such as digital inclusive finance (Peng and Mao, 2023; Liu and Guo, 2023), social security expenditures (Yu and Li, 2021), agricultural cooperatives (Gava et al., 2021; Shen et al., 2022), and land transfer (Li F. et al., 2023; Liu et al., 2024) and household characteristics and behaviors e.g., non-agricultural employment (Naminse and Zhuang, 2018), training participation (Wan et al., 2021), and human capital (Su and Guo, 2022). While external factors are significant, the effective alleviation of relative poverty fundamentally depends on individuals’ autonomy and self-awareness; otherwise, we risk a scenario best described as “the spearhead does not move, but the spear pole is overtired to break.”

Based on the aforementioned analysis, we used rural sample data from the 2018 China Family Panel Studies (CFPS) to empirically examine the impact of non-cognitive abilities on the relative poverty of farming households. This study makes three potential contributions. First, we integrated non-cognitive abilities into the traditional economic model framework and developed a theoretical framework to analyze their effects on farming households’ relative poverty, particularly in terms of optimizing household economic decision-making. This approach offers theoretical insights into establishing a sustainable mechanism for alleviating relative poverty. Second, we explored the causality between non-cognitive abilities and relative poverty among farming households and attempted to uncover the underlying mechanisms of this relationship, thereby shedding more light on the influence of non-cognitive abilities on relative poverty. Third, we assessed the effectiveness of non-cognitive abilities in reducing relative poverty by focusing on the relative poverty gap, rather than merely the status of relative poverty.

## 2 Theoretical analysis and research hypotheses

### 2.1 Non-cognitive ability and the alleviation of farmer households’ relative poverty

The “rational man” hypothesis, a foundational assumption of microeconomics regarding human behavioral choices, is increasingly challenged by empirical evidence suggesting that individuals are not entirely rational but are influenced by non-cognitive abilities when making economic decisions (Heckman et al., 2006). These abilities significantly impact the quality of economic decision-making in farmer households, which in turn affects their income levels and relative poverty (Luan and Bauer, 2016). Economic decision-making is primarily exhibited through labor in economic life, and non-cognitive abilities can influence income levels by affecting labor productivity (Bowles et al., 2001).

Almlund et al. (2011) proposed that labor productivity is influenced by two types of factors: (1) endowment characteristics, such as human capital attributes (e.g., education level); and (2) effort levels, encompassing the time and effort expended. While endowment characteristics are relatively stable, effort levels are dictated by subjective attitudes and willingness. With consistent endowment characteristics, a higher effort level leads to greater labor productivity. Farmer households with robust non-cognitive abilities—characterized by traits such as enterprising spirit, aggressiveness, willingness to cooperate, social skills, and optimism—demonstrate a strong drive for self-development and are less likely to exhibit passive behaviors (such as waiting, reliance, and wanting). Consequently, they tend to invest more time and effort in agricultural and non-agricultural production (Jia et al., 2023), achieving higher labor productivity and increased income, and consequently alleviating relative poverty.

Additionally, non-cognitive abilities optimize the selection of livelihood strategies for farmer households. For instance, innovative and curious households are more likely to adopt advanced agricultural technologies, boosting the valuation of their agrarian yields while elevating business revenue (Abay et al., 2017). Similarly, enterprising and aggressive households are more likely to pursue entrepreneurial activities, augmenting their operating income. In summary, Families demonstrating elevated non-cognitive abilities exhibit a reduced propensity toward encountering relative poverty. Therefore, this study proposes Hypothesis l: improving non-cognitive abilities can alleviate relative poverty among farmer households.

### 2.2 Underlying mechanism for the alleviating effect of non-cognitive ability on farmer households’ relative poverty

Conventional economics argues that individuals’ economic decisions are primarily influenced by constraints, preferences, and expectations, and are typically determined exogenously. However, evidence suggests a clear connection between personality traits (non-cognitive abilities) and these three major factors influencing economic decisions (Brocklebank et al., 2011). Non-cognitive abilities shape personal limitations through bolstering the capacity to acquire knowledge, facilitate asset building, and cultivate competency progression. Individuals with high non-cognitive abilities gain access to a broader array of choices and resources, facilitating more effective decision-making (Borghans et al., 2008).

According to bounded rationality theory, decision-makers are limited by information-processing ability, and farmers often rely on empirical rules to make judgments. Non-cognitive ability with information-screening ability and critical thinking enable active searching for information and reduce the degree of information asymmetry. In the digital economy era, marked by advancements such as 5G, big data, and artificial intelligence, the Internet has become a vital tool. It provides farmer households with enhanced access to information, improves the quality of information obtained, and amplifies their information acquisition capabilities (Li F. et al., 2023). Given the resource scarcity in rural areas (Kabir et al., 2020), the Internet’s convenience and low cost significantly improve these households’ access to information. Additionally, the Internet offers abundant educational and training resources (Xie H. et al., 2023), which can be leveraged by farmer households with high non-cognitive abilities to enhance their human capital, thereby increasing their income levels and alleviating relative poverty.

In addition, social capital is an important way for farmers to obtain information. Farmers with social initiative and empathy can break through the traditional closed social network with family and local ties as the core, and actively embed new organizational forms, such as cooperatives and e-commerce alliances. Moreover, farmers who are good communicators are more likely to establish a trust relationship with scientific research institutions, take the lead in mastering advanced technology for agricultural production, and improve total factor productivity. The enrichment of social capital not only brings instant access to resources, but more importantly, it forms a continuous information exchange channel, so that farmers can dynamically adjust their production and management strategies and effectively deal with the relative poverty risk brought by market fluctuations.

Preferences reflect the tendencies and attitudes individuals exhibit when faced with various choices; these are subjective psychological factors evident in economic decision-making and align closely with non-cognitive abilities (Almlund et al., 2011). Once basic survival needs are met, enterprising and aggressive farmer households are likely to utilize various support policies to expand production or venture into non-agricultural activities, adapting to the market economy and improving their income levels. As inclusive finance improves in quality and effectiveness, households with high non-cognitive abilities are more inclined to secure funding through formal financial institutions and decrease their aversion to financial instruments (Xie S. et al., 2023), thereby obtaining the necessary funds to expand production scales or engage in entrepreneurial ventures (Jiang and Fan, 2022). This significantly increases their income.

Expectations involve the choices that individuals make based on objective information in anticipation of future uncertainties. Limited by information asymmetry and resource scarcity, traditional farmers often show excessive risk aversion. Farmers with strong non-cognitive ability usually have more optimistic emotions, stronger risk tolerance, and learning ability, and can rationally evaluate the long-term benefits of existing production conditions, break through the “status quo bias” trap, and thus, have positive expectations for the future development of individuals. Such positivity implies that these households will work and live more diligently, face challenges and setbacks with optimism, and thus contribute to the alleviation of relative poverty. Therefore, this study proposes Hypothesis 2: non-cognitive abilities can enhance the quality of household economic decision-making by relieving constraints, adjusting preferences, and improving expectations, thereby alleviating the relative poverty of farmer households.

## 3 Materials and methods

### 3.1 Data sources

**Figure 1:**
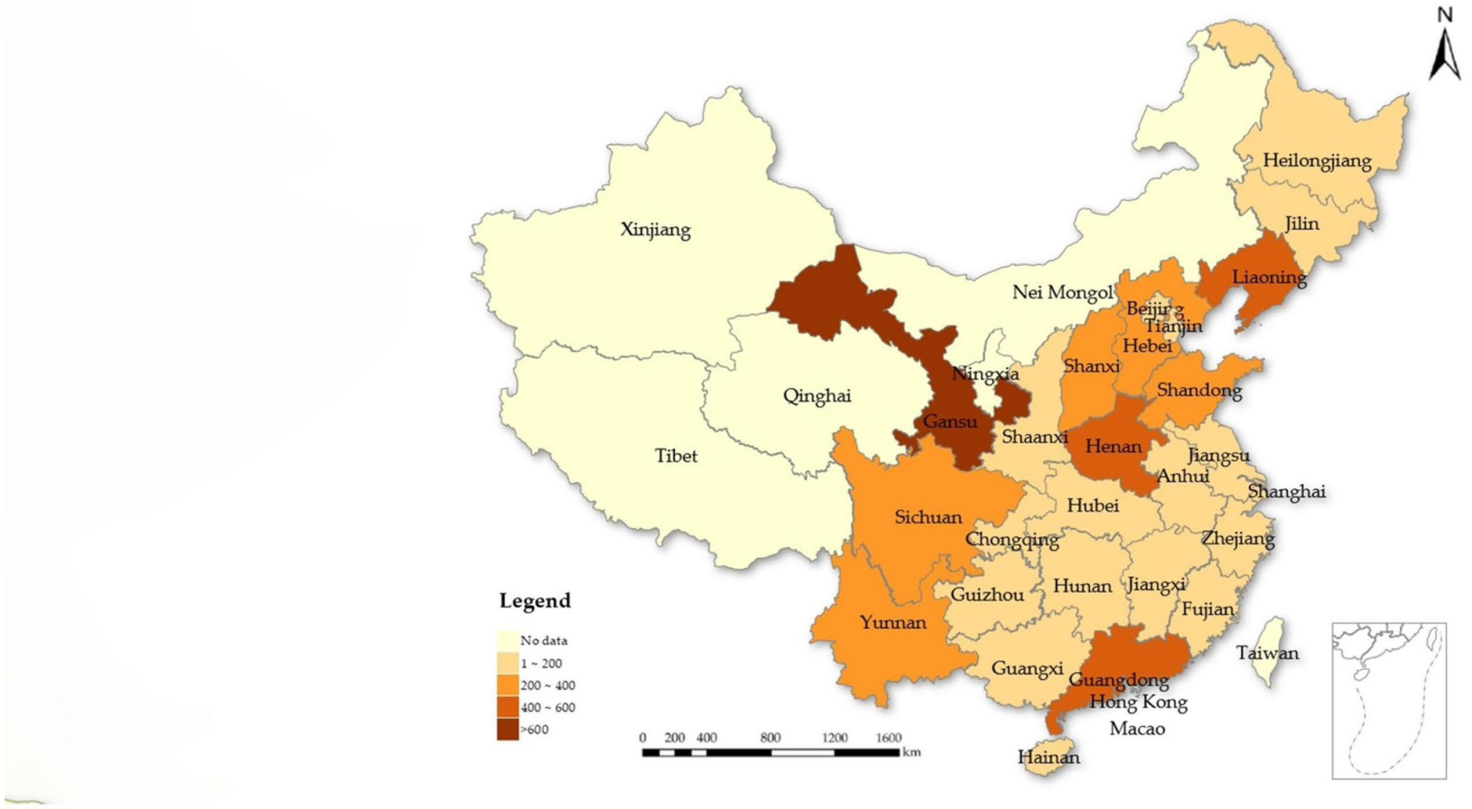
The spatial distribution of sample provinces. The map is based on standard of China No. GS (2023) 2767.

The data for this study were sourced from the rural sample collected through the CFPS in 2018. The CFPS was designed to capture socio-economic changes across China and included follow-up surveys of representative samples from 25 provincial regions (Xie and Hu, 2014). As shown in Figure 1, the sample data do not cover the provinces of Xinjiang, Tibet, Qinghai, Nei Mongol, Ningxia, Taiwan, Hong Kong, and Macao. The provinces of Gansu, Guangdong, Liaoning, and Henan are more representative, with Gansu having the largest sample size of over 600 households. CFPS data are highly regarded for their representativeness and have been extensively utilized by researchers. For this study, we specifically chose the 2018 CFPS rural sample because it provided the relevant statistics on non-cognitive abilities. We focused on assessing the impact of non-cognitive abilities on the relative poverty of farmer households. We primarily drew information on non-cognitive abilities from the adult questionnaire survey results, while the assessment of relative poverty among these households was based on the household questionnaire survey results (Xu and Xie, 2017). Given the pivotal role of household heads in economic decision-making and significant household matters, the non-cognitive abilities of these individuals were used to represent the non-cognitive abilities of the farmer households. During data processing, we matched the data from the two types of questionnaires using household codes and excluded samples with missing key variables. Ultimately, this process yielded a total of 5,123 observations for the study.

### 3.2 Variable selection

#### 3.2.1 Core explanatory variables

##### 3.2.1.1 Non-cognitive ability

It is primarily measured using three methods: personality tests, questionnaire surveys, and behavioral experiments, with questionnaire surveys being the most common approach. The Big Five Personality Traits method is the most widely accepted method for such surveys (Costa and McCrae, 1992). To minimize differences and hierarchies between specific dimensions of non-cognitive ability, this method was chosen to measure non-cognitive ability in our study. The Big Five personality traits—conscientiousness, extroversion, agreeableness, openness, and emotional stability—form a five-dimensional non-cognitive ability index system. The CFPS provided questions associated with these five dimensions (Table 1). Each dimension is evaluated using three questions, with each question offering five response options ranging from “1: totally disagree” to “5: totally agree.” Given that indicators under different dimensions may vary in their relative contributions to non-cognitive ability, the coefficient of variation method was utilized to measure these indicators. This objective weighting approach allocates significance to individual indicators by evaluating the variation extent between existing and desired outcomes. A high degree of dispersion suggests that an indicator is far from reaching its target value, warranting a higher weight; conversely, a lower weight is assigned when dispersion is minimal. In this study, the weight of each indicator within a dimension is expressed as $w_{ij}=v_{ij}÷\overset{3}{\underset{j=1}{\sum }}v_{ij}$ , where $v_{ij}$ denotes the coefficient of variation for the j -th indicator under the i -th dimension. Using these weights, we calculated the sub-indicators for each of the five dimensions. Each dimension’s weight was then determined using its coefficient of variation: $w_{ij}=v_{i}÷\overset{5}{\underset{i=1}{\sum }}v_{i}$ , where $v_{i}$ and $w_{ij}$ denote the coefficient of variation and the weight of the i -th dimension, respectively. The overall non-cognitive ability indicator was then calculated based on the weight assigned to each dimension. It is important to note that indicators such as “usually lazy” under conscientiousness, “reserved and conservative” under extroversion, “sometimes rude and unkind to others” under agreeableness, and “often worried” and “easily nervous” under emotional stability are considered contrary indicators and require reverse adjustments. The reversed scoring for these indicators is “1: totally disagree,” “2: disagree,” “3: neither agree nor disagree,” “4: agree,” and “5: totally agree.” The adjusted overall non-cognitive ability indicator and sub-indicators for each dimension are all aligned in a positive direction.

**Table 1:** Questions associated with the big five personality traits in the CFPS.

Big five personality traits	Key features	Corresponding questions in the CFPS
Conscientiousness	Reflects an individual’s sense of responsibility and level of effort.	Rigorous and conscientious Usually lazy Work efficiently
Extraversion	Reflects an individual’s decisiveness, aggressiveness, and activeness.	Talkative Cheerful and sociable Reserved and conservative
Agreeableness	Reflects an individual’s readiness to cooperate with others, their tolerance, and their trust in others.	Sometimes rude and unkind to others Tolerant by nature Considerate and kind to almost everyone
Openness	Reflects an individual’s creativity, innovativeness, and curiosity.	Creative and innovative Values artistic and aesthetic experiences Imaginative
Emotional stability	Reflects an individual’s confidence, optimism, and resistance to stress.	Often worried Easily nervous Relaxed and copes with pressure easily

#### 3.2.2 Explained variables

##### 3.2.2.1 Farmer households’ relative poverty

Relative poverty describes a condition where individuals’ income or consumption levels fall below a certain threshold compared to the societal average, typically defined as 40 to 60% of median income (Saunders, 2007; Qi and Wu, 2016). As China progresses from eliminating absolute poverty to addressing relative poverty, establishing the poverty threshold at 40% of rural residents’ median disposable income proves methodologically sound. Statistical records from 2018 indicate the median disposable income for rural inhabitants reached 13,066 yuan nationally, which positions the relative poverty benchmark at 5,226 yuan based on this standardized calculation framework.

To effectively assess the degree of relative poverty alleviation, the relative poverty gap is used as the dependent variable in benchmark regression. Additionally, the relative poverty gap rate and relative poverty status are employed as dependent variables in robustness tests. Specifically, if a farmer household’s per-capita net income falls below the relative poverty line, the relative poverty gap is calculated as the difference between the relative poverty line and the household’s per-capita net income. The relative poverty gap rate is then determined by dividing this difference by the relative poverty line. When a farmer household’s per-capita net income equals or exceeds the relative poverty line, both the relative poverty gap and relative poverty gap rate are zero.

#### 3.2.3 Mechanism variables

##### 3.2.3.1 Constraint variable

The “frequency of using the Internet to learn” is used to measure constraints. This variable is set to 0 when the respondent indicates “never” for using the Internet for learning purposes or not using the Internet at all. If the respondent uses the Internet to learn at any frequency, the value is set to 1. When any adult member of a farmer household participates in learning via the Internet, the household is considered to use the Internet for learning, and the value is set to 1.

##### 3.2.3.2 Preference variable

The “preferred lender” item in the survey questionnaire reflects farmer households’ borrowing channel preferences. Values are assigned as follows: if the respondent selects “parents or children,” “relatives,” or “friends,” the value is 1, indicating a preference for borrowing from acquaintances. A choice of “private lending institutions and individuals” is assigned a value of 2, suggesting a preference for private lenders. The option “never borrow money under any circumstances” is assigned a value of 3, indicating no clear borrowing preference. Selections of “non-bank formal financial institutions” and “banks” are given values of 4 and 5, respectively, indicating a preference for formal lending channels.

##### 3.2.3.3 Expectation variable

“Confidence in your future” is gauged on a scale from 1 to 5, ranging from “no confidence” to “very confident.” This variable is designed to capture the respondent’s expectations about their future, with higher values indicating more positive expectations.

#### 3.2.4 Control variables

Control variables in this study encompass both household-head-level variables and household-level variables, which are classic determinants of farmer households’ income and relative poverty. Household-head-level variables include age, gender, educational level, health, and marital status. At the household level, variables such as household size, dependency ratio, and government subsidies are considered. To address potential omitted variable bias, the quadratic term of the household head’s age is also included in the regression analysis. Additionally, cognitive ability, which influences both non-cognitive ability and relative poverty, is treated as a control variable. The household head’s cognitive ability is assessed through scores in mathematical and literacy modules. The mathematical module consists of 24 questions, and the literacy module contains 34 questions, arranged in increasing order of difficulty. The score for each module is determined by the number of the most difficult questions answered correctly.

Table 2 displays the descriptive statistics for key variables. The mean value of the relative poverty gap is 0.145, and that of the relative poverty gap rate is 0.017. The mean value of the relative poverty status is 0.225, indicating that 22.5% of the farmer households in the sample are classified as experiencing relative poverty. As for non-cognitive ability, the overall mean value is 3.307. Among its five dimensions, conscientiousness scores the highest with a mean value of 3.896, followed by agreeableness, extroversion, and openness. Emotional stability scores the lowest, with a mean value of 2.984.

**Table 2:** Descriptive statistics of key variables.

Variable	Definition	Mean	Variance	Max	Min
Relative poverty gap	(Relative poverty line) - (household per-capita net income) (logarithm)	0.145	0.484	0	8.561
Relative poverty gap rate	[(Relative poverty line) - (household per-capita net income)] / (relative poverty line)	0.017	0.057	0	1
Relative poverty status	1: Household per-capita net income is below the relative poverty line; 0: otherwise	0.225	0.418	0	1
Non-cognitive ability	Overall non-cognitive ability indicator	3.307	0.399	1.726	4.736
Conscientiousness	Non-cognitive ability sub-indicator	3.896	0.648	1	5
Extraversion	Non-cognitive ability sub-indicator	3.393	0.701	1	5
Agreeableness	Non-cognitive ability sub-indicator	3.819	0.608	1	5
Openness	Non-cognitive ability sub-indicator	3.119	0.890	1	5
Emotional stability	Non-cognitive ability sub-indicator	2.984	0.740	1	5
Household head’s age	Unit: year	53.675	13.326	16	92
Gender	1: male; 0: female	0.554	0.497	0	1
Educational level	Number of years of education	6.129	4.173	0	16
Health	5: very healthy; 4: healthy; 3: fairly healthy; 2: averagely healthy; 1: unhealthy	2.774	1.281	1	5
Marital status	1: married; 0: otherwise	0.857	0.350	0	1
Household size	Total number of household members	3.952	1.990	1	21
Dependency ratio	(Number of underage and elderly household members) / (total household size)	0.440	0.311	0	1
Governmental subsidy	1: Available government subsidy; 0: otherwise	0.630	0.483	0	1
Mathematical ability	Score of the mathematical module, measuring the cognitive ability	6.787	4.285	0	24
Literacy ability	Score of the literacy module, measuring the cognitive ability	16.214	10.117	0	34
Internet learning	1: yes; 0: no	0.348	0.477	0	1
Lending channel preference	1: borrow from acquaintance; 2: private lending; 3: no obvious preference; 4: non-bank financial institutions; 5; banks	2.253	1.688	1	5
Confidence in the future	Values 1 to 5 indicate “unconfident” to “very confident”	4.113	1.025	1	5

### 3.3 Model settings

This study investigates the impact of non-cognitive ability on the relative poverty of farmer households. The empirical model is formulated as follows (Equation 1):


(1) $$\mathrm{Rpga}p_{jh}=\alpha_{0}+\alpha_{1}\mathrm{Noncognitiv}e_{jh}+\underset{1}{\sum }\alpha_{i}X_{jh}+\delta_{j}+\varepsilon_{jh},i>1$$

where, Rpgap denotes the relative poverty gap and Noncognitive represents the non-cognitive ability; j denotes the j -th provincial region, h denotes the h -th farmer household, X represents a series of control variables, δ indicates the provincial fixed effect, and ε is the random error term. Standard errors are clustered at the household level.

Equation 2 is designed for testing mechanisms and evaluates how non-cognitive ability alleviates relative poverty by influencing farmer households’ constraints, preferences, and expectations. It is expressed as follows:


(2) $$\mathrm{Me}d_{jh}=\beta_{0}+\beta_{0}\mathrm{Noncognitiv}e_{jh}+\underset{1}{\sum }\beta_{i}X_{jh}+\delta_{j}+\mu_{jh},i>1$$

where, Med denotes the mechanism variables including constraints, preferences, and expectations represented by the importance of the Internet as an information channel, borrowing channel preference, and confidence in the future, respectively. μ is the random error term, and Noncognitive , j , h , X , and δ retain their meanings from Model (1).

## 4 Results

### 4.1 Benchmark regression results

Column (1) of Table 3 displays the results of the OLS estimation for Model (1). The estimated coefficient for non-cognitive ability is significantly negative, indicating that improvements in non-cognitive ability help alleviate relative poverty among farmer households. Column (2) explores the heterogeneous effects of the five sub-indicators of non-cognitive ability on relative poverty. Owing to some collinearity among these sub-indicators, Columns (3) to (7) each analyze the impact of different dimensions of non-cognitive ability on relative poverty. The results reveal that the coefficients for conscientiousness and extroversion are significantly negative. This suggests that households with high levels of concentration, efficiency, methodicalness, sense of responsibility, effort, decisiveness, and aggressiveness are less likely to experience relative poverty. Conversely, agreeableness, openness, and emotional stability fail to demonstrate a statistically meaningful association with the relative poverty of farmer households.

**Table 3:** Benchmark regression results of non-cognitive ability and relative poverty in farmer households.

Variable	Relative poverty gap							
	(1)	(2)	(3)	(4)	(5)	(6)	(7)	(8)
Non-cognitive ability	—0.029**							—0.264***
	(0.014)							(0.070)
Conscientiousness		—0.016	—0.019*					
		(0.011)	(0.010)					
Extraversion		—0.018*		—0.020**				
		(0.009)		(0.009)				
Agreeableness		—0.008			—0.014			
		(0.009)			(0.009)			
Openness		0.011				0.009		
		(0.008)				(0.008)		
Emotional stability		—0.007					—0.013	
		(0.009)					(0.009)	
Household head’s age	—0.018***	—0.017***	—0.017***	—0.018***	—0.018***	—0.018***	—0.018***	—0.016***
	(0.005)	(0.005)	(0.005)	(0.005)	(0.005)	(0.005)	(0.005)	(0.005)
The quadratic term of the head’s age	0.000***	0.000***	0.000***	0.000***	0.000***	0.000***	0.000***	0.000***
	(0.000)	(0.000)	(0.000)	(0.000)	(0.000)	(0.000)	(0.000)	(0.000)
Gender	0.008	0.005	0.005	0.006	0.004	0.005	0.009	0.023
	(0.014)	(0.015)	(0.014)	(0.014)	(0.014)	(0.014)	(0.015)	(0.015)
Educational level	—0.007***	—0.007***	—0.008***	—0.007***	—0.008***	—0.008***	—0.007***	—0.006***
	(0.002)	(0.002)	(0.002)	(0.002)	(0.002)	(0.002)	(0.002)	(0.002)
Health	—0.012**	—0.011**	—0.012**	—0.012**	—0.013**	—0.014**	—0.012**	0.002
	(0.006)	(0.006)	(0.006)	(0.006)	(0.006)	(0.006)	(0.006)	(0.007)
Marital status	—0.050*	—0.049*	—0.049*	—0.050*	—0.051*	—0.051*	—0.050*	—0.045*
	(0.026)	(0.026)	(0.027)	(0.027)	(0.026)	(0.026)	(0.026)	(0.026)
Household size	0.004	0.005	0.005	0.005	0.005	0.005	0.004	0.004
	(0.004)	(0.004)	(0.004)	(0.004)	(0.004)	(0.004)	(0.004)	(0.004)
Dependency ratio	0.077**	0.079**	0.077**	0.077**	0.078**	0.077**	0.076**	0.077**
	(0.032)	(0.032)	(0.032)	(0.032)	(0.032)	(0.032)	(0.032)	(0.032)
Governmental subsidy	—0.044**	—0.044**	—0.045**	—0.044**	—0.044**	—0.045***	—0.045***	—0.043**
	(0.017)	(0.017)	(0.017)	(0.017)	(0.017)	(0.017)	(0.017)	(0.017)
Mathematical ability	—0.001	—0.001	—0.001	—0.001	—0.001	—0.001	—0.001	—0.001
	(0.002)	(0.002)	(0.002)	(0.002)	(0.002)	(0.002)	(0.002)	(0.002)
Literacy ability	—0.004***	—0.003***	—0.004***	—0.004***	—0.004***	—0.004***	—0.004***	—0.003**
	(0.001)	(0.001)	(0.001)	(0.001)	(0.001)	(0.001)	(0.001)	(0.001)
Provincial fixed effect	Yes	Yes	Yes	Yes	Yes	Yes	Yes	Yes
N	5,123	5,123	5,123	5,123	5,123	5,123	5,123	5,123
DWH-ꭓ2 statistic								8.329***
First-stage F-statistic								33.650***
KP rk LM statistic								254.476***

Standard errors for clustering at the household level are in parentheses. ***, **, and * indicate significance at the 1, 5, and 10% levels, respectively.

Although non-cognitive ability is significantly negatively correlated with the relative poverty of farmer households, there are compelling reasons to believe that the non-cognitive ability of households experiencing high degrees of relative poverty might be adversely affected by various factors such as shortages of material resources, poor health, and under-education. This suggests the potential for an endogeneity problem due to reverse causality. To mitigate the influence of endogeneity on the empirical findings, we used the mean value of non-cognitive ability within districts or counties as an instrumental variable. Interactions among farmer households within these areas can influence their non-cognitive abilities; thus, the district-or county-level non-cognitive ability may impact the non-cognitive ability of individual households. However, it does not directly affect the relative poverty of these households, thereby fulfilling the basic requirements for an instrumental variable.

Column (8) of Table 3 displays the regression results using the two-stage least squares (2SLS) method. The Durbin–Wu–Hausman (DWH) test indicates that non-cognitive ability is an endogenous explanatory variable, and the coefficient estimates from Column (1) are biased. The first-stage F-statistic of 33.65 exceeds the commonly used threshold of 10, confirming the relevance of the instrumental variable. Additionally, the Kleibergen-Paap rk LM statistic is 254.476, significantly above the critical value of 16.38 at the 10% error level, indicating that there is no issue of weak instrumental variables. Compared to the results in Column (1), the estimated coefficient for non-cognitive ability remains significantly negative at the 1% statistical level in the 2SLS regression, but the marginal effect is slightly higher. This finding suggests that failing to account for endogeneity leads to an underestimation of the impact of non-cognitive ability. When endogeneity is considered, an increase of one standard deviation in the overall non-cognitive ability indicator leads to a 10.53% decrease in farmer households’ relative poverty. Therefore, Hypothesis 1 is supported by the estimation results.

### 4.2 Heterogeneity analysis

#### 4.2.1 Regional heterogeneity

Columns (1) and (2) of Table 4 present the regression results for farmer households in Western China and in Eastern and Central China, respectively. Evidently, non-cognitive ability is significantly negatively correlated with relative poverty in both regions. However, the impact is more pronounced in Western China. An increase of one standard deviation in overall non-cognitive ability results in a 16.72% decrease in the relative poverty gap for farmer households in Western China, indicating a substantial alleviating effect. Compared to their counterparts in Eastern and Central China, farmer households in Western China tend to exhibit traits of closed-mindedness and passiveness, which contribute to conservative and outdated production and development patterns. This conservatism hinders their ability to escape poverty and achieve wealth. However, as non-cognitive abilities improve and old mindsets are abandoned, farmer households in Western China begin to adopt agricultural and non-agricultural livelihood strategies similar to those in Eastern and Central China. Consequently, they experience more significant income growth and a more substantial alleviation of relative poverty than those in the more developed regions.

**Table 4:** Non-cognitive ability and relative poverty in farmer households: regional heterogeneity, gender heterogeneity, and age heterogeneity.

Variable	Western China	Eastern and Central China	Female-headed	Male-headed	Old generation	New generation
	(1)	(2)	(3)	(4)	(5)	(6)
Non-cognitive ability	—0.419***	—0.165*	—0.305**	—0.233***	—1.119***	—0.874***
	(0.121)	(0.087)	(0.121)	(0.084)	(0.310)	(0.185)
Control variables	Yes	Yes	Yes	Yes	Yes	Yes
Provincial fixed effect	Yes	Yes	Yes	Yes	Yes	Yes
N	1,827	3,296	2,285	2,838	3,951	1,172
DWH-ꭓ2 statistic	7.348***	4.057**	3.536*	5.045**	5.222**	5.101**
first-stage F-statistic	22.140***	17.750***	11.130***	10.630***	18.490***	17.310***
KP rk LM statistic	109.066***	145.182***	120.342***	142.326***	157.700***	39.229***

Standard errors for clustering at the household level are in parentheses. ***, **, and * indicate significance at the 1, 5, and 10% levels, respectively.

#### 4.2.2 Gender heterogeneity

Columns (3) and (4) of Table 4 present the regression results for female-headed and male-headed farmer households, respectively. The results indicate that non-cognitive ability is significantly negatively correlated with relative poverty in both groups. However, the alleviation of relative poverty is more significant among female-headed households. An increase of one standard deviation in overall non-cognitive ability results in a 12.17% decrease in the relative poverty gap for female-headed farmer households, indicating a substantial alleviating effect. Compared to men, women often face disadvantages in terms of risk exposure, market competitiveness, and social skills (Chen and Zhang, 2024). Consequently, while improvements in non-cognitive ability do not produce a significant marginal effect among male-headed households, which already have comparative advantages, they can substantially bridge these gaps for women. In female-headed households, enhancements in non-cognitive ability can significantly increase income levels (Li H. et al., 2023), thereby alleviating their relative poverty more effectively.

#### 4.2.3 Age heterogeneity

Columns (5) and (6) of Table 4 present the regression results for the old generation (age > 45 years) and the new generation (age < = 45 years) farmer households, respectively. The results indicate that non-cognitive ability is significantly negatively correlated with relative poverty in both groups. However, the alleviation of relative poverty is more significant among old generation households. The old generation relies more on social relations to maintain living resources than the new generation does. Social skills and emotional management skills in non-cognitive abilities can help them maintain intergenerational support and effectively make up for the lack of economic capital.

#### 4.2.4 Educational heterogeneity

The sample is divided into five groups according to the education level of the head of the household: non-school, primary school, junior high school, senior high school, university and above. The regression results presented in Table 5 show that the effect of non-cognitive ability on alleviating the relative poverty of farmers is significant for the non-school, primary school, and junior high school groups, but is not significant for the senior high school and university and above groups. Farmer households in the non-school, primary school, and junior high school groups are primarily involved in manual labor or low-end services. In these sectors, traits such as high conscientiousness and emotional stability contribute to better focus and improved labor productivity, while extroversion, agreeableness, and openness enhance performance and yield higher returns in low-end service industries. Conversely, farmer households in the high-educational-level group are typically employed in more knowledge-and technology-oriented jobs, where non-cognitive abilities play a limited role in boosting income and alleviating poverty less effectively compared to the low-educational-level group. This disparity suggests a complementary relationship between non-cognitive abilities and higher educational levels.

**Table 5:** Non-cognitive ability and relative poverty in farmer households: educational heterogeneity.

Variable	Non-school	Primary school	Junior high school	Senior high school	University and above
	(1)	(2)	(3)	(4)	(5)
Non-cognitive ability	—0.853***	—1.250***	—1.118***	—0.568	—0.022
	(0.286)	(0.441)	(0.264)	(0.363)	(0.726)
Control variables	Yes	Yes	Yes	Yes	Yes
Provincial fixed effect	Yes	Yes	Yes	Yes	Yes
N	1,252	1,489	1,759	500	123
DWH-ꭓ2 statistic	6.338***	5.750**	3.638*	5.045**	3.672**
first-stage F-statistic	17.300***	15.190***	12.890***	22.540***	19.780***
KP rk LM statistic	72.489***	40.401***	66.675***	22.797***	16.362**

Standard errors for clustering at the household level are in parentheses. ***, **, and * indicate significance at the 1, 5, and 10% levels, respectively.

It is noteworthy that the “timely rain” role of non-cognitive ability in alleviating relative poverty, particularly among female-headed farmer households, low-educated farmer households, farmer households in western China, and non-school, primary school, and junior high school farmer households, highlights its inclusive nature.

### 4.3 Robustness test

#### 4.3.1 Replacing the explained variable

In line with the analyses in the previous sections, the relative poverty gap was replaced with the relative poverty gap rate and relative poverty status to reassess the alleviating effect of non-cognitive ability on farmer households’ relative poverty using Model (1). Columns (1) and (2) of Table 6 display the estimation results. The results of the Wald and DWH tests confirm that non-cognitive ability is an endogenous explanatory variable. Additionally, the results of the first-stage F-statistic and Kleibergen-Paap rk LM statistic indicate that there is no issue with weak instrumental variables. Furthermore, the estimated coefficients for non-cognitive ability in both columns are significantly negative, suggesting that improvements in non-cognitive ability contribute to reducing not only the relative poverty gap rate but also the likelihood of falling into relative poverty. Thus, we can conclude that non-cognitive ability alleviates farmer households’ relative poverty.

**Table 6:** Non-cognitive ability and relative poverty in farmer households: robustness test.

Variable	Relative poverty status	Relative poverty gap rate	Non-cognitive ability	Relative poverty gap	Non-cognitive ability	Relative poverty gap 2020	Relative poverty gap 2022
	IV-Probit	2SLS	2SLS	2SLS	IV-Tobit	2SLS	2SLS
	(1)	(2)	(3)	(4)	(5)	(6)	(7)
Non-cognitive ability	—1.283^***^	—0.031^***^	—0.048^***^		—1.593^***^	—0.910^***^	—0.852^***^
	(0.203)	(0.008)	(0.014)		(0.342)	(0.160)	(0.151)
New non-cognitive ability				—0.162^***^			
				(0.044)			
Control variables	Yes	Yes	Yes	Yes	Yes	Yes	Yes
Provincial fixed effect	Yes	Yes	Yes	Yes	Yes	Yes	Yes
N	5,123	5,123	5,123	5,123	5,123	5,123	5,123
Wald/DWH-ꭓ2 statistic	25.260^***^	8.329^***^	8.244^***^	14.364^***^	22.420^***^	27.681^***^	14.364^***^
first-stage F-statistic		33.650^***^	32.070^***^	133.200^***^		254.480^***^	539.650^***^
KP rk LM statistic		254.476^***^	318.084^***^	113.391^***^		192.161^***^	192.161^***^

Standard errors for clustering at the household level are in parentheses. ***, **, and * indicate significance at the 1, 5, and 10% levels, respectively.

#### 4.3.2 Re-measuring non-cognitive ability

To refine our measurement, the variable for each dimension of non-cognitive ability was calculated by averaging the scores of three questions within each dimension. Subsequently, the overall non-cognitive ability indicator was derived by summing these measurement variables across all five dimensions. This revised approach was used to re-estimate the impact of non-cognitive ability on farmer households’ relative poverty. The estimation results are presented in Column (3) of Table 6. The DWH test results confirm that non-cognitive ability is an endogenous explanatory variable. Furthermore, the first-stage *F*-value and Kleibergen-Paap rk LM statistic demonstrate that there is no issue with weak instrumental variables. Although the magnitude of the re-measured regression coefficient for non-cognitive ability differs from that reported previously, it still shows a significantly negative correlation with relative poverty. Thus, our primary conclusion—that non-cognitive ability alleviates farmer households’ relative poverty—remains unchanged, despite variations in the weighting of the non-cognitive ability measurement indicator.

In addition, using the Revised NEO Personality Inventory questionnaire as the classification basis, the British Family Panel Studies, and the German Socio-economic Panel Studies, this study looks for self-evaluation and other-evaluation data that can measure non-cognitive ability from the CFPS questionnaire, and uses principal component analysis to measure new non-cognitive ability. The regression results are shown in Column (4) of Table 6. After replacing the non-cognitive ability index, non-cognitive ability still significantly slows down the relative poverty of farmers at the 1% statistical level, and the estimation results of the benchmark regression are robust.

#### 4.3.3 Changing the estimation method

Given that the sample exclusively contained relatively impoverished farmer households, we also applied the IV-Tobit method for coefficient estimation when using the relative poverty gap as the measure of relative poverty. The estimation results are displayed in Column (5) of Table 6. The results of the Wald test confirm that non-cognitive ability is an endogenous explanatory variable. Moreover, the estimated coefficient for non-cognitive ability is significantly negative at the 1% statistical level. This finding indicates that even when employing the IV-Tobit method, our conclusion remains consistent: non-cognitive ability significantly alleviates relative poverty among farmer households.

#### 4.3.4 The long-term impact of non-cognitive ability on farmers ‘relative poverty

To explore the long-term impact of non-cognitive ability on farmers’ relative poverty, this study regresses the explanatory variable non-cognitive ability lagged by one period and two periods. The regression results are shown in Column (6) and (7) of Table 6. The regression coefficients of non-cognitive ability lagged by one period and two periods are significantly negative, indicating that non-cognitive ability can slow down the relative poverty of farmers in the long run.

## 5 Mechanism analysis of how non-cognitive ability alleviates farmers’ relative poverty

This section examines how non-cognitive ability alleviates the relative poverty of farmer households by relieving constraints, adjusting preferences, and improving expectations. Empirical evidence has shown that farmer households can utilize the Internet to overcome poverty and increase income (Zhou et al., 2020), and access funds through formal lending channels to boost income (Wang et al., 2018) and enhance future confidence (Jumiyati, 2019). Therefore, this analysis focuses on the impact of non-cognitive ability on Internet usage for learning, preferences for lending channels, and confidence in future prospects.

### 5.1 Relieving constraints

Using the IV-Tobit method, we conducted coefficient estimation for Model (2) to explore the causality between non-cognitive ability and Internet learning and between non-cognitive ability and Internet social networking. The estimation results, presented in Columns (1)–(4) of Table 7, demonstrate that the coefficients of non-cognitive ability are significantly positive. This indicates that improvements in non-cognitive ability increase the likelihood of farmer households using the Internet for learning. Utilizing the Internet for information acquisition alleviates constraints related to resource shortages and poor transportation, effectively preventing farmer households from falling into the “digital divide.” It helps them make more informed economic decisions and engage more actively in market activities by reducing information asymmetry. Furthermore, Internet social networking allows farmer households to acquire vocational skills tailored to their needs, compensating for deficiencies in human capital, enhancing their employment competitiveness, and increasing their labor remuneration. Evidently, non-cognitive ability can stimulate farmer households to earn more through Internet learning, thus alleviating their relative poverty.

**Table 7:** The mechanism of non-cognitive ability affecting relative poverty in farmer households: relieving constraints.

Variable	Internet learning		Internet social networking		Social capital	
	IV-Probit	IV-Probit	IV-Probit	IV-Probit	2SLS	2SLS
	(1)	(2)	(3)	(4)	(5)	(6)
Non-cognitive ability	0.533^**^	0.089^**^	0.472^*^	0.855^***^	0.308^*^	0.059^*^
	(0.209)	(0.040)	(0.253)	(0.288)	(0.176)	(0.034)
Control variables	Yes	Yes	Yes	Yes	Yes	Yes
Provincial fixed effect	Yes	Yes	Yes	Yes	Yes	Yes
N	5,123	5,123	5,123	5,123	5,123	5,123
Wald/DWH-ꭓ2 statistic	2.950^*^	2.770^*^	5.853^**^	4.454^**^	5.548^**^	6.569^**^
first-stage F-statistic					283.010^***^	319.840^***^
KP rk LM statistic					207.212^***^	232.943^***^

Standard errors for clustering at the household level are in parentheses. ***, **, and * indicate significance at the 1, 5, and 10% levels, respectively. (1), (3), and (5) are listed as regression results for non-cognitive ability as measured by the coefficient of variation method, and (2), (4), and (6) are listed as regression results for non-cognitive ability as measured by summation at the time of the robustness test.

Columns (5) and (6) of Table 7 list the 2SLS estimation results of non-cognitive ability on farmers’ social capital. The results show that the influence of non-cognitive ability measured by the two methods on farmers’ social capital is significantly positive at the statistical level of 5%. Extroversion in non-cognitive ability is the key for farmers to break through the limitations of traditional geographical relations. Individuals with strong social initiative are more likely to participate in village-level affairs, non-agricultural activities, and agricultural exchanges, which not only expands the social radius of individuals, but also accesses heterogeneous resources through weak relationship networks, thus breaking through the homogenization limitations of strong relationship networks, which helps alleviate farmers’ relative poverty.

### 5.2 Adjusting preferences

Columns (1) and (2) of Table 8 detail the results from the 2SLS estimation of how non-cognitive ability affects farmer households’ preferences for lending channels. The results, which are significant at the 5% statistical level for both methods, indicate that an improvement in non-cognitive ability enhances farmer households’ preference for formal lending channels. With the deepening implementation of inclusive finance—a key strategy for promoting rural revitalization in rural China—the supply-side credit constraints on farmer households are being gradually alleviated. This shift toward formal financial institutions helps relieve demand-side credit constraints, thereby providing farmer households with sufficient funds for production development. Most Chinese farmer households are risk-averse and tend to allocate available funds to production development under the dual pressures of enhancing production and avoiding potential credit defaults, leading to income generation. From the perspective of the intensive margin, obtaining more funds allows for the expansion of existing agricultural production scales. From the extensive margin, accessing more capital promotes entrepreneurial activities. Through both approaches, enhancing lending channel preferences can alleviate relative poverty by increasing household operational income.

**Table 8:** The mechanism of non-cognitive ability affecting relative poverty in farmer households: adjusting preferences and improving expectations.

Variable	Preferences for lending channels		Level of confidence in the future	
	2SLS	2SLS	2SLS	2SLS
	(1)	(2)	(3)	(4)
Non-cognitive ability	0.696^**^	0.109^**^	0.897^***^	0.184^***^
	(0.293)	(0.055)	(0.170)	(0.031)
Control variables	Yes	Yes	Yes	Yes
Provincial fixed effect	Yes	Yes	Yes	Yes
N	5,123	5,123	5,123	5,123
Wald/DWH-ꭓ^2^ statistic	6.301^**^	4.814^**^	5.962^**^	6.069^**^
first-stage F-statistic	33.650^***^	32.070^***^	33.650^***^	32.070^***^
KP rk LM statistic	254.476^***^	318.084^***^	254.476^***^	318.084^***^

Standard errors for clustering at the household level are in parentheses. ***, **, and * indicate significance at the 1, 5, and 10% levels, respectively. (1), and (3) are listed as regression results for non-cognitive ability as measured by the coefficient of variation method, and (2), and (4) are listed as regression results for non-cognitive ability as measured by summation at the time of the robustness test.

### 5.3 Improving expectations

Poverty manifests in two primary forms: material poverty and mental poverty. Typically, those experiencing material poverty also suffer from mental poverty, characterized by a lack of confidence and ambition. This mental state, in turn, can exacerbate material poverty, creating a cyclical interaction between the two (Helminiak, 2020). Mental poverty often stems from a lack of motivation for self-development. Therefore, improving non-cognitive abilities can reduce the likelihood of falling into mental poverty. Columns (3) and (4) of Table 8 demonstrate that the estimated coefficient values for non-cognitive ability, as measured by both methods, are significantly positive. This indicates that enhancing non-cognitive abilities can significantly boost farmer households’ confidence in their future. Consequently, a high level of non-cognitive ability elevates expectations for the future, motivating households to overcome poverty and achieve prosperity. This shift instills the belief that “a good life is achieved through struggle,” prompting households to adopt more proactive approaches in production and daily life, which in turn improves their income levels and alleviates relative poverty.

In summary, non-cognitive ability can optimize farmer households’ decision-making by relieving constraints, adjusting preferences, and improving expectations. This includes giving greater importance to Internet learning, increasing the preference for formal lending channels, and boosting confidence in the future, all of which contribute to alleviating their relative poverty. Thus, Hypothesis 2 is confirmed.

## 6 Conclusion

Studying the cognitive aspects of pro-social behavior to understand the psychological processes that drive individuals to participate in actions that are beneficial to others or the whole society, is conducive to forming a cognitive mechanism that enhances the well-being of others and strengthens social connections and social identity (Li et al., 2019). By combining cognitive science with pro-social behavior research, this paper aims to reveal the psychological process of promoting altruism, empathy, and social cohesion. This interdisciplinary approach not only enriches the academic understanding of human behavior and social interaction theory but also provides practical insights for the formulation of effective social policies and interventions. It is of great significance to explore the cognitive factors that affect the development of pro-social tendencies in different cultures, social environments, and individual differences. The contemporary phase following poverty eradication has witnessed rural families’ income disparity emerging as a critical socioeconomic challenge, drawing significant academic and public focus regarding sustainable development strategies. To date, limited scholarship has explored the effects of self-improvement capacities on relative poverty from an individual and household perspective. In this study, we incorporated non-cognitive abilities into the traditional economic model framework to empirically investigate their causal effect on farmer households’ relative poverty.

First, non-cognitive ability is significantly negatively correlated with farmer households’ relative poverty. Specifically, an increase of one standard deviation in non-cognitive ability leads to a 10.53% decrease in relative poverty. Among the five dimensions of non-cognitive ability, conscientiousness and extraversion are significantly negatively correlated with relative poverty, whereas agreeableness, openness, and emotional stability do not have a significant impact.

Second, compared to high-educated and male-headed farmer households and those in Eastern and Central China, high non-cognitive ability more significantly alleviates relative poverty among low-educated and female-headed farmer households and those in Western China, thereby playing a “timely rain” role and demonstrating inclusiveness.

Third, non-cognitive ability enhances the quality of household economic decision-making by promoting Internet learning, increasing preferences for formal financial institutions, and boosting future confidence, thus alleviating relative poverty. Access to information and the enhancement of human capital can contribute to higher levels of household income and alleviate relative poverty. Farmers with high non-cognitive skills can adapt to the market economy and alleviate credit constraints better, which in turn alleviates relative poverty. Farmers with positive expectations are able to deal with challenges and setbacks better, helping to alleviate relative poverty.

This study also has reference significance for other countries in efforts to alleviate relative poverty. Developing countries have the core features of a small-scale peasant economy and family labor force. Regardless of whether farmers are rice growers in Southeast Asia, mixed farming families in Africa, or small coffee producers in Latin America, their production decisions are limited by land fragmentation, shortage of funds, and market information asymmetry. This commonality lends universal value to the risk decision-making ability and resource integration ability in non-cognitive ability. The characteristics of the family as the basic economic unit make the mode of action of non-cognitive traits, such as responsibility and patience, in the transmission of intergenerational human capital cross-culturally stable. Our research results show that improving non-cognitive ability can enhance the ability of information collection, resource accumulation, and skill improvement, and help farmers obtain more economic resources, thus alleviating relative poverty. Therefore, the conclusions of this study not only provide solid evidence for non-cognitive ability to alleviate relative poverty, but also enable governments to better recognize the important role of non-cognitive ability in alleviating relative poverty.

## 7 Research limitations

First, the China Family Panel Studies only investigated issues related to non-cognitive abilities in 2018; thus, this article could only use cross-sectional data for analysis. However, cross-sectional analysis cannot reflect the long-term impact of non-cognitive ability on farmers’ relative poverty. Moreover, many potential factors (e.g., genetic characteristics, early living environment) are difficult to measure completely at a single time point, and missing variables may cause bias in the estimation results of this study. Future research should use panel data to analyze this issue.

Second, the construction of non-cognitive ability indicators uses the Big Five personality classification method, which cannot capture all the characteristics of non-cognitive ability. Pro-social behavior is also an important part of non-cognitive ability. The lack of a pro-social behavior dimension in the construction of non-cognitive ability indicators may lead to measurement errors. Future research should add pro-social behavior dimensions.

Third, this paper uses China as an example to analyze the impact of non-cognitive ability on the relative poverty of farmers. Whether the results are applicable to other countries remains to be verified.

## Data Availability

Publicly available datasets were analyzed in this study. This data can be found at: https://opendata.pku.edu.cn/dataverse/CFPS.

## References

[ref1] Abay K. A. Blalock G. Berhane G. (2017). Locus of control and technology adoption in developing country agriculture: evidence from Ethiopia. J. Econ. Behav. Organ. 143, 98–115. doi: 10.1016/j.jebo.2017.09.012

[ref2] Almlund M. Duckworth A. L. Heckman J. Kautz T. (2011). Personality psychology and economics. Handb. Econ. Educ. 4, 1–181. doi: 10.1016/B978-0-444-53444-6.00001-8

[ref3] Anghel B. Balart P. (2017). Non-cognitive skills and individual earnings: new evidence from PIAAC. SERIEs. 8, 417–473. doi: 10.1007/s13209-017-0165-x

[ref4] Borghans L. Duckworth A. L. Heckman J. J. Ter Weel B. (2008). The economics and psychology of personality traits. J. Hum. Resour. 43, 972–1059. doi: 10.1353/jhr.2008.0017

[ref5] Bowles S. Gintis H. Osborne M. (2001). The determinants of earnings: a behavioral approach. J. Econ. Lit. 39, 1137–1176. doi: 10.1257/jel.39.4.1137

[ref6] Brocklebank S. Lewis G. J. Bates T. C. (2011). Personality accounts for stable preferences and expectations across a range of simple games. Pers. Individ. Dif. 51, 881–886. doi: 10.1016/j.paid.2011.07.007

[ref7] Buser T. Ahlskog R. Johannesson M. Koellinger P. Oskarsson S. (2024). The causal effect of genetic variants linked to cognitive and non-cognitive skills on education and labor market outcomes. Lab. Econ. 90:102544. doi: 10.1016/j.labeco.2024.102544

[ref8] Chen Y. Zhang X. (2024). Gender differences in relation of gender role attitudes and happiness—a mixed-methods research from China. Front. Psychol. 15:1419942. doi: 10.3389/fpsyg.2024.1419942, PMID: 39346500 PMC11427413

[ref9] Costa P. T. McCrae R. R. Jr. (1992). Four ways five factors are basic. Pers. Individ. Dif. 13, 653–665. doi: 10.1016/0191-8869(92)90236-I

[ref10] Gava O. Ardakani Z. Delalić A. Azzi N. Bartolini F. (2021). Agricultural cooperatives contributing to the alleviation of rural poverty. The case of Konjic (Bosnia and Herzegovina). J. Rural. Stud. 82, 328–339. doi: 10.1016/j.jrurstud.2021.01.034

[ref11] Heckman J. J. Stixrud J. Urzua S. (2006). The effects of cognitive and noncognitive abilities on labor market outcomes and social behavior. J. Lab. Econ. 24, 411–482. doi: 10.1086/504455

[ref12] Helminiak D. A. (2020). Material and spiritual poverty: a postmodern psychological perspective on a perennial problem. J. Relig. Health 59, 1458–1480. doi: 10.1007/s10943-019-00873-z, PMID: 31317467

[ref13] Jia H. Sai X. Si H. Wang J. (2023). How do the non-cognitive skills affect retirees’ reemployment? Evidence from China. Front. Public Health. 11:1128241. doi: 10.3389/fpubh.2023.1128241, PMID: 38169704 PMC10758448

[ref14] Jiang R. Fan W. (2022). Inclusive finance and employment: can financial development improve peasant’s entrepreneurship? Manag. Decis. Econ. 43, 630–646. doi: 10.1002/mde.3407

[ref15] Jumiyati S. (2019). Increasing income of cocoa farming through the role of agricultural extension and strengthening institutional capacity of farmers. Int. J. Agric. Environ. Biores. 4, 110–121. doi: 10.35410/IJAEB.2019.4465

[ref16] Kabir M. H. Azad M. J. Islam M. N. (2020). Exploring the determinants and constraints of smallholder vegetable farmers’ adaptation capacity to climate change: a case of Bogura District. Bangladesh. J. Agric. Crop Res. 8, 176–186. doi: 10.33495/jacr_v8i9.20.159

[ref17] Levine R. Rubinstein Y. (2017). Smart and illicit: who becomes an entrepreneur and do they earn more? Q. J. Econ. 132, 963–1018. doi: 10.1093/qje/qjw044

[ref18] Li H. (2024). The influence of noncognitive ability on the wage of rural migrant workers. Int. J. 45, 639–660. doi: 10.1108/IJM-03-2023-0128

[ref19] Li H. Chen C. Zhang Z. (2023). Are gender differences related to non-cognitive abilities?——evidence from China. J. Asia Pac. Econ. 28, 1560–1579. doi: 10.1080/13547860.2021.1982483

[ref20] Li F. He K. Zhu R. Zhang J. Gao M. (2023). Rural low-carbon energy development in the information age: can internet access drive the farmer to participate in personal carbon trading schemes related to bioenergy? Sustain. Dev. 31, 1417–1432. doi: 10.1002/sd.2456

[ref21] Li J. Li A. Sun Y. Li H. E. Liu L. Zhan Y.. (2019). The effect of preceding self-control on prosocial behaviors: the moderating role of awe. Front. Psychol. 10:682. doi: 10.3389/fpsyg.2019.00682, PMID: 30971994 PMC6443925

[ref22] Li J. Zhan Y. Fan W. Liu L. Li M. Sun Y.. (2018). Sociality mental modes modulate the processing of advice-giving: an event-related potentials study. Front. Psychol. 9:42. doi: 10.3389/fpsyg.2018.00042, PMID: 29467689 PMC5808223

[ref23] Liu L. Guo L. (2023). Digital financial inclusion, income inequality, and vulnerability to relative poverty. Soc. Indic. Res. 170, 1155–1181. doi: 10.1007/s11205-023-03245-z

[ref24] Liu S. Xu H. Deng L. (2024). Does land transfer help alleviate relative poverty in China? An analysis based on income and capability perspective. Appl. Econ. 57, 723–735. doi: 10.1080/00036846.2024.2305619

[ref25] Luan D. X. Bauer S. (2016). Does credit access affect household income homogeneously across different groups of credit recipients? Evidence from rural Vietnam. J. Rural. Stud. 47, 186–203. doi: 10.1016/j.jrurstud.2016.08.001

[ref26] Meng Y. Lu Y. Liang X. (2024). Does internet use alleviate the relative poverty of Chinese rural residents? A case from China. Environ. Dev. Sustain. 26, 11817–11846. doi: 10.1007/s10668-023-03531-3, PMID: 40078838

[ref27] Naminse E. Y. Zhuang J. (2018). Does farmer entrepreneurship alleviate rural poverty in China? Evidence from Guangxi Province. Plos One 13:e0194912. doi: 10.1371/journal.pone.0194912, PMID: 29596517 PMC5875809

[ref28] Palczyńska M. (2021). Wage premia for skills: the complementarity of cognitive and non-cognitive skills. Int. J. Manpow. 42, 556–580. doi: 10.1108/IJM-08-2019-0379

[ref29] Parise G. Peijnenburg K. (2019). Noncognitive abilities and financial distress: evidence from a representative household panel. Rev. Financ. Stud. 32, 3884–3919. doi: 10.1093/rfs/hhz010

[ref30] Peng P. Mao H. (2023). The effect of digital financial inclusion on relative poverty among urban households: a case study on China. Soc. Indic. Res. 165, 377–407. doi: 10.1007/s11205-022-03019-z

[ref31] Qi D. Wu Y. (2016). Child income poverty levels and trends in urban China from 1989 to 2011. Child Indic. Res. 9, 1043–1058. doi: 10.1007/s12187-015-9351-1

[ref32] Ramkissoon H. (2020). Place confinement, pro-social, pro-environmental behaviors, and residents’ wellbeing: a new conceptual framework. Front. Psychol. 11:2248. doi: 10.3389/fpsyg.2020.02248, PMID: 32982895 PMC7490327

[ref33] Saunders P. (2007). Comparing poverty among older people in urban China internationally. China Q. 190, 451–465. doi: 10.1017/S0305741007001282

[ref34] Shen Y. Wang J. Wang L. Wu B. Ye X. Han Y.. (2022). How do cooperatives alleviate poverty of farmers? Evidence from rural China. Land. 11:1836. doi: 10.3390/land11101836, PMID: 40053772

[ref35] Smithers L. G. Sawyer A. C. P. Chittleborough C. R. Davies N. M. Davey Smith G. Lynch J. W. (2018). A systematic review and meta-analysis of effects of early life non-cognitive skills on academic, psychosocial, cognitive and health outcomes. Nat. Hum. Behav. 2, 867–880. doi: 10.1038/s41562-018-0461-x, PMID: 30525112 PMC6277013

[ref36] Su J. Guo S. (2022). Human capital and rural households’ vulnerability to relative poverty: evidence from China. Discret. Dyn. Nat. Soc. 2022:3960691. doi: 10.1155/2022/3960691

[ref37] Trajano S. S. Sousa-Filho J. M. D. Matos S. Lessa B. S. (2023). Do volunteers intend to become social entrepreneurs? The influence of pro-social behavior on social entrepreneurial intentions. Nonprofit Volunt. Sect. Q. 52, 443–473. doi: 10.1177/08997640221103299

[ref38] Wan G. Hu X. Liu W. (2021). China’s poverty reduction miracle and relative poverty: focusing on the roles of growth and inequality. China Econ. Rev. 68:101643. doi: 10.1016/j.chieco.2021.101643

[ref39] Wang S. Cao P. Huang S. (2022). Household financial literacy and relative poverty: an analysis of the psychology of poverty and market participation. Front. Psychol. 13:898486. doi: 10.3389/fpsyg.2022.898486, PMID: 35936289 PMC9355557

[ref40] Wang X. Chen M. He X. Zhang F. (2018). Credit constraint, credit adjustment, and sustainable growth of farmers’ income. Sustain. For. 10:4407. doi: 10.3390/su10124407

[ref41] Xie Y. Hu J. (2014). An introduction to the China family panel studies (CFPS). Chin. Sociol. Rev. 47, 3–29. doi: 10.2753/CSA2162-0555470101.2014.11082908

[ref42] Xie S. Jin C. Song T. Feng C. (2023). Research on the long tail mechanism of digital finance alleviating the relative poverty of rural households. PLoS One 18:e0284988. doi: 10.1371/journal.pone.0284988, PMID: 37104485 PMC10138262

[ref43] Xie H. Zhang J. Shao J. (2023). Difference in the influence of internet use on the relative poverty among farmers with different income structures. Econ. Anal. Policy 78, 561–570. doi: 10.1016/j.eap.2023.03.022

[ref44] Xu H. Xie Y. (2017). Socioeconomic inequalities in health in China: a reassessment with data from the 2010-2012 China family panel studies. Soc. Indic. Res. 132, 219–239. doi: 10.1007/s11205-016-1244-2, PMID: 28694561 PMC5501396

[ref45] Yi S. Huo Z. Zhang M. Chen F. (2023). An empirical study of new rural collective economic organization in alleviating relative poverty among farmers. Sustain. For. 15:14126. doi: 10.3390/su151914126

[ref46] Yu L. R. Li X. Y. (2021). The effects of social security expenditure on reducing income inequality and rural poverty in China. J. Integr. Agric. 20, 1060–1067. doi: 10.1016/S2095-3119(20)63404-9

[ref47] Zhou X. Cui Y. Zhang S. (2020). Internet use and rural residents’ income growth. China Agric. Econ. Rev. 12, 315–327. doi: 10.1108/CAER-06-2019-0094

